# Fibronectin Adherent Cell Populations Derived From Avascular and Vascular Regions of the Meniscus Have Enhanced Clonogenicity and Differentiation Potential Under Physioxia

**DOI:** 10.3389/fbioe.2021.789621

**Published:** 2022-01-28

**Authors:** Girish Pattappa, Franziska Reischl, Judith Jahns, Ruth Schewior, Siegmund Lang, Johannes Zellner, Brian Johnstone, Denitsa Docheva, Peter Angele

**Affiliations:** ^1^ Laboratory for Experimental Trauma Surgery, Department of Trauma Surgery, University Regensburg Medical Centre, Regensburg, Germany; ^2^ Sporthopaedicum Regensburg, Regensburg, Germany; ^3^ Department of Orthopaedics and Rehabilitation, Oregon Health and Science University, Portland, OR, United States; ^4^ Department of Musculoskeletal Tissue Regeneration, Orthopaedic Hospital König-Ludwig-Haus, University of Wurzburg, Wurzburg, Germany

**Keywords:** meniscus, hypoxia, tissue engineeering, meniscus progenitor cells, chondrogenesis

## Abstract

The meniscus is composed of an avascular inner region and vascular outer region. The vascular region has been shown to contain a progenitor population with multilineage differentiation capacity. Strategies facilitating the isolation and propagation of these progenitors can be used to develop cell-based meniscal therapies. Differential adhesion to fibronectin has been used to isolate progenitor populations from cartilage, while low oxygen or physioxia (2% oxygen) enhances the meniscal phenotype. This study aimed to isolate progenitor populations from the avascular and vascular meniscus using differential fibronectin adherence and examine their clonogenicity and differentiation potential under hyperoxia (20% oxygen) and physioxia (2% oxygen). Human vascular and avascular meniscus cells were seeded onto fibronectin-coated dishes for a short period and monitored for colony formation under either hyperoxia or physioxia. Non-fibronectin adherent meniscus cells were also expanded under both oxygen tension. Individual fibronectin adherent colonies were isolated and further expanded, until approximately ten population doublings (passage 3), whereby they underwent chondrogenic, osteogenic, and adipogenic differentiation. Physioxia enhances clonogenicity of vascular and avascular meniscus cells on plastic or fibronectin-coated plates. Combined differential fibronectin adhesion and physioxia isolated a progenitor population from both meniscus regions with trilineage differentiation potential compared to equivalent hyperoxia progenitors. Physioxia isolated progenitors had a significantly enhanced meniscus matrix content without the presence of collagen X. These results demonstrate that combined physioxia and fibronectin adherence can isolate and propagate a meniscus progenitor population that can potentially be used to treat meniscal tears or defects.

## Introduction

The menisci are located on the medial and lateral tibial plateau of the knee joint that aids in load bearing, force transmission (e.g., compression, tension, and shear), and lubrication during joint motion. It also protects the underlying articular cartilage from high impact forces that can induce cartilage lesions and result in early osteoarthritis (Makris et al., 2011; Madry et al., 2016; Verdonk et al., 2016). The meniscus is a fibrocartilaginous tissue that has two distinct regions: an inner avascular region and an outer vascularized region. The avascular region resembles articular cartilage with a high proportion of collagen type II and glycosaminoglycans, while the vascular region of the meniscus contains a higher proportion of collagen type I (Ghadially et al., 1983; McDevitt and Webber, 1990; Verdonk et al., 2005). Meniscal tears or lesions are a common injury with a high annual incidence (66 per 100,000) throughout the world (reviewed by Bansal et al., 2021). Patients with meniscal tears undergo partial or total meniscectomy, but this leads to exposure of the underlying cartilage, resulting in altered joint biomechanics that increases risk of developing early osteoarthritis (Englund et al., 2009; Paradowski et al., 2016). Nowadays, clinicians have begun to preserve the meniscus, although the capacity for repair depends on the localization of the injury. Specifically, tears in the vascular region can be healed by applying sutures, whilst tears in the avascular region have a limited repair capacity, partially due to the low vascularity in this part of the meniscus. Methods to treat meniscus tears are required to prevent further damage to the meniscus and underlying tissues, thus preventing the onset of early osteoarthritis.

Cell-based treatment strategies for meniscus repair have focused on treating the avascular region of the tissue. Studies using mesenchymal stromal cells (MSCs) have shown their superior regenerative properties compared to meniscal cells (Pabbruwe et al., 2010; Nakagawa et al., 2015; Whitehouse et al., 2017; Zellner et al., 2017). The use of these cells in patients has also resulted in improved clinical outcomes (Whitehouse et al., 2017; Olivos-Meza et al., 2019; Sekiya et al., 2019). However, studies using MSCs have demonstrated the induction of cartilage hypertrophy that upon *in vivo* implantation, leads to ectopic bone formation (Pelttari et al., 2006). Furthermore, many countries only permit the use of homologous approaches for treating tissues, which limits the use of MSCs from various tissue sources for clinical treatments.

Previous studies have demonstrated that the meniscus from both animal (e.g., bovine and rabbit) and human sources contains a progenitor population with the ability to differentiate to the adipogenic, osteogenic, and chondrogenic lineage (Mauck et al., 2007; Muhammad et al., 2014; Huang et al., 2016; Gamer et al., 2017; Seol et al., 2017; Chahla et al., 2021; Wei et al., 2021). Specifically, these progenitors were mainly located in the vascular region and horns of the meniscus (Mauck et al., 2007). Muhammad et al. (2014) and Seol et al. (2017) showed that a meniscus progenitor cell population can also be isolated *via* cell migration from meniscus explants derived from bovine or human tissues. The former study showed that these progenitor populations were derived from the superficial layer of the meniscus (Muhammad et al., 2014). A recent investigation has also demonstrated the presence of progenitor population within the avascular meniscus regions (Chahla et al., 2021).

A method to isolate progenitor populations is the use of differential adhesion to fibronectin. Fibronectin is present in both the stem cell niche and meniscus tissues (Salter et al., 1995). It enables selection of progenitor populations due to the presence of alpha-5/beta-1 integrin on their cell surface (Dowthwaite et al., 2004). Previous investigations have isolated multipotent progenitors from skin and bone marrow using this technique (Jones and Watt, 1993; D'Ippolito et al., 2004; Williams et al., 2010). Articular cartilage progenitor cells (ACPs) isolated *via* fibronectin adhesion were found to express stem cell markers (e.g., CD90, CD105, and Notch), increased telomerase activity, and have multilineage differentiation potential. In contrast to MSCs, use of ACPs in an animal model resulted in cartilage regeneration with no evidence of bone formation (Dowthwaite et al., 2004; Williams et al., 2010). Furthermore, a recent study has shown that fibronectin adherent populations exist in osteoarthritic meniscus tissue that are clonogenic, have a high proliferation capacity, express MSC surface markers and maintain differentiation potential at high passages compared to non-fibronectin adherent meniscus cells (Korpershoek et al., 2021)**.** Thus, differential fibronectin adherence has the potential to isolate an appropriate cell type from the tissue that can be used for meniscus treatment or regeneration.

*In vivo*, the meniscus is under a low oxygen environment or physioxia (2%–5% oxygen) similar to articular cartilage (Lund-Olesen, 1970; Ivanovic, 2009; Lafont, 2010; Ivanovic and Vlaski, 2015). In general, physioxia has shown a donor-dependent enhancement in cartilage-related gene expression and matrix content for chondrocytes and chondrogenic MSCs under physioxia compared to normal oxygen tension (20% oxygen) or hyperoxia (Pattappa et al., 2019b). In the case of meniscus cells, studies have also demonstrated that physioxia has a beneficial effect on their gene expression and matrix content (Adesida et al., 2006; Adesida et al., 2007; Adesida et al., 2012; Berneel et al., 2016; Liang et al., 2017; Szojka et al., 2021). Studies examining stem cells also indicate that low oxygen tension helps maintain their stemness and extends their proliferation capacity compared to normal oxygen tension (D'Ippolito et al., 2006).

The present investigation combined the use of differential fibronectin adhesion and physioxic culture to investigate the presence of progenitor cells from avascular and vascular regions of human meniscus. Isolated progenitor populations expanded under hyperoxia and physioxia, subsequently underwent differentiation to the adipogenic, osteogenic and chondrogenic lineage at passage 2 (≈ 10 population doublings). In-depth analysis of chondrogenic differentiation was investigated to determine the matrix-forming capacity of these progenitors and their potential in treating meniscus tears. In parallel, experiments were replicated for non-fibronectin adherent (NFA) avascular and vascular meniscus cells. Based on previous investigations, it was hypothesized that fibronectin facilitated the isolation of a meniscus progenitor population and that physioxia has a beneficial effect on the clonogenicity and differentiation potential of these cells.

## Materials and Methods

### Meniscus Cell and Fibronectin Isolation

**FIGURE 1:**
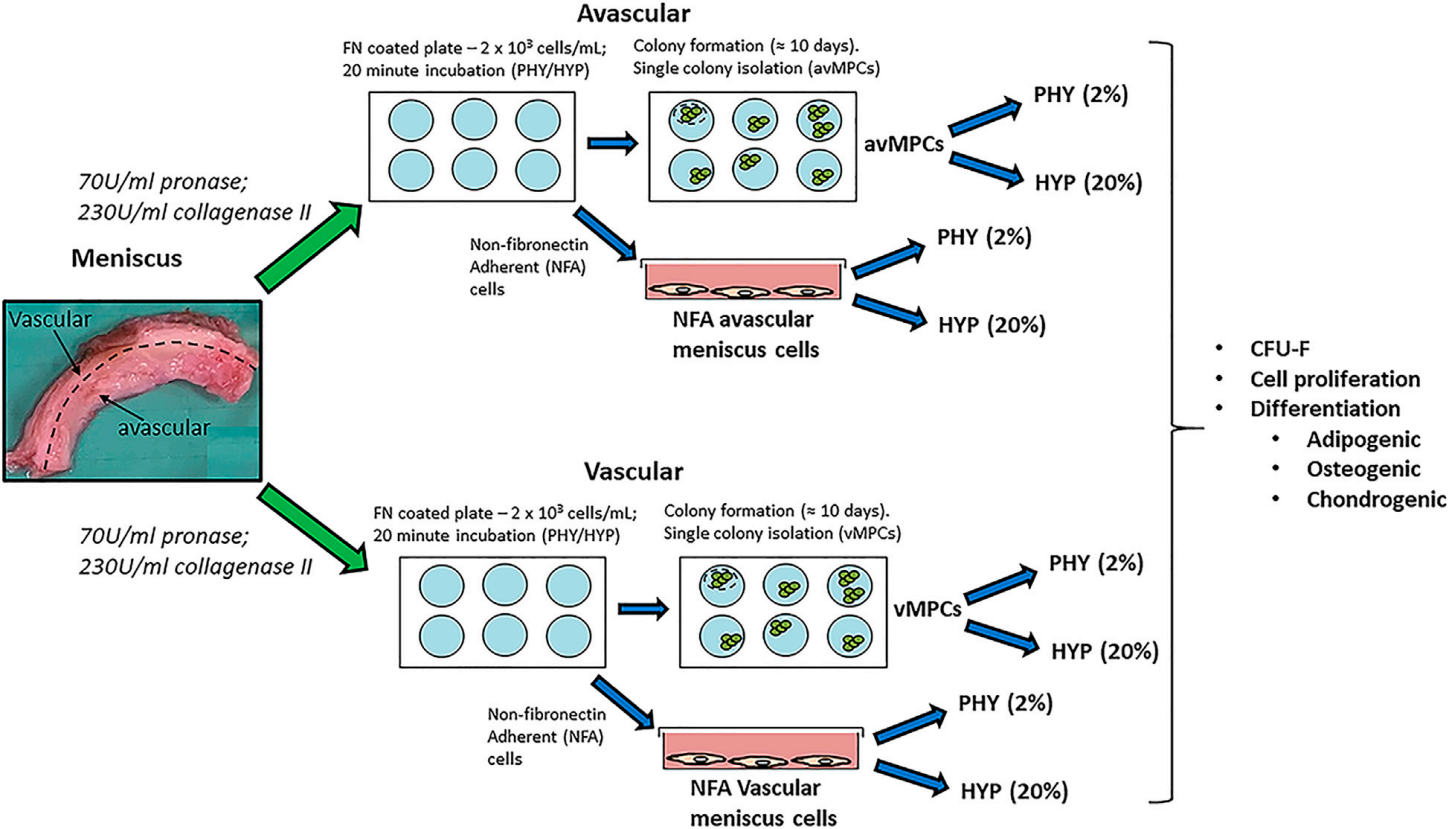
Schematic describing the experimental plan for non-fibronectin adherent (NFA) avascular and vascular meniscus cells and isolation of fibronectin adherent progenitors (avMPCs and vMPCs) expanded and differentiated under hyperoxia (HYP/20% oxygen) and physioxia (PHY/2% oxygen).

Human meniscus tissue was collected from patients (*n* = 7; mean age: 60 ± 7) with no previous meniscus injury and undergoing total knee arthroplasty, following informed consent from patients and using procedures approved by the local ethics committee (University Hospital Regensburg; Ethic approval no.: Nr. 18-837-101). Meniscus tissue was split into its two regions (inner two-thirds for the avascular region and outer one-third for the vascular region), minced into small pieces and digested in pronase (70 U/ml; Roche Diagnostics, Switzerland) in DMEM-LG (Invitrogen, Karlsruhe, Germany) + 5% FBS (PAN-Biotech, Aidenbach, Germany) + 1% penicillin-streptomycin (Sigma-Aldrich, Steinheim, Germany) for 45 min and then overnight in collagenase type II (230 U/ml; Worthington) in DMEM-LG + 5% FBS + 1% penicillin-streptomycin at 37°C (Figure 1).

For the fibronectin adherence, six-well plates were coated with 10 mg/ml bovine fibronectin (Sigma-Aldrich, Steinheim, Germany) in PBS with magnesium and calcium chloride ions (Sigma-Aldrich, Steinheim, Germany) and incubated overnight at 4°C. Following collagenase digestion, the number of avascular and vascular meniscus cells was counted using a hemacytometer, then each cell type was seeded at 2 × 10^3^ cells/ml (200 cells/cm^2^) onto fibronectin-coated plates and incubated for 20 min in either a standard cell culture incubator at 20% oxygen and 5% CO_2_ or a low oxygen incubator (ThermoFisher Scientific, Regensburg Germany) set at 2% oxygen and 5% CO_2_. For this manuscript, hyperoxia refers to 20% oxygen, while 2% oxygen is termed as physioxia. Following a 20-min incubation period, media containing non-fibronectin adherent meniscus cells was removed and replaced with expansion media composed of DMEM + 10% FBS + 5 ng/ml basic fibroblast growth factor (Peprotech, Hamburg, Germany) + 1 ng/ml TGF-beta1 (R&D Systems, Oxford, UK). Non-fibronectin adherent avascular and vascular meniscus cells were seeded at 5 × 10^3^ cells/cm^2^ with equal numbers of flasks placed under both oxygen tensions and cultured using the same the expansion media. Media changes were performed twice per week using pre-equilibrated media at the same oxygen tension. Upon observation of colonies, individual meniscus progenitor cell colonies were isolated as previously described (Williams et al., 2010). Three clones were evaluated for each meniscus region under their respective oxygen tension. Henceforth, clones isolated *via* fibronectin are either avascular fibronectin adherent meniscus progenitors (avMPCs) or vascular fibronectin adherent meniscus progenitor (vMPCs) cells. Non-fibronectin adherent (NFA) avascular and vascular meniscus cells, avMPCs, and vMPCs were expanded for 50 days and cell counts were recorded for population growth curve using previously described calculations (Pattappa et al., 2020).

### Colony-Forming Unit Assay (CFU-F)

Colony-forming unit (CFU) assay was performed on P1 NFA meniscus cells from both oxygen conditions and on uncoated and fibronectin-coated dishes. In brief, 10-cm Petri dishes were seeded with NFA avascular or vascular meniscus cells at 20, 5, and 2 cells/cm^2^ with a set of dishes also coated with 10 mg/ml fibronectin as described above. After 10 days of cultivation, formed colonies were stained using 0.5% crystal violet/methanol and imaged (Sigma, Steinheim, Germany), and individual colonies were counted as previously described (Kohler et al., 2013; Alberton et al., 2015).

### Differentiation

All differentiations were performed on passage 2 (≈10 population doublings) NFA avascular and vascular meniscus cells, avMPCs, and vMPCs under their respective expansion oxygen conditions using previously described protocols (Alberton et al., 2015).

### Osteogenic Differentiation and Alizarin Red Staining

Cells were seeded at 2 × 10^3^ cells/cm^2^ into 12-well plates and incubated under their respective expansion oxygen conditions. Following 24 h after seeding, media was replaced with osteogenic medium [DMEM-HG (Invitrogen, Karlsruhe, Germany) + 10% FBS + 0.05 mM L-ascorbic acid-2-phosphate + 10 mM *β*-glycerophosphate + 0.1 mM dexamethasone (all Sigma-Aldrich, Steinheim, Germany)] and cultured for 21 days with media refreshed twice per week. For controls, cells were cultured In DMEM-HG + 10% FBS + 1% penicillin-streptomycin for the duration of culture. At 21 days of culture, calcium deposition was visualized using alizarin red staining using previously described protocols (Alberton et al., 2015). Monolayers were imaged using an inverted microscope (Nikon, Japan).

### Adipogenic Differentiation and Oil-Red-O Staining

Cells were seeded at 5 × 10^3^ cells/cm^2^ into a 24-well plate and then incubated in expansion media till 80% confluence. Media was then replaced with adipogenic induction media composed of DMEM-HG (Invitrogen) + 10% FBS + 1% penicillin-streptomycin + 0.1 mg/ml insulin (PAN Biotech, Aidenbach, Germany) + 1 μM dexamethasone + 0.2 mM indomethacin + 0.1 mg/ml insulin + 1 mM 3-isobutyl-1-methylxanthine (all Sigma Aldrich, Steinheim, Germany). Control cultures were cultured in adipogenic maintenance media composed of DMEM-HG + 10% FBS + 1% penicillin-streptomycin + 0.1 mg/ml insulin. Cells were stimulated for 21 days with a week cycle for adipogenic cultures consisting of 5 days adipogenic induction media and 2 days of adipogenic maintenance media. Lipid vacuoles were visualized by Oil-Red-O staining by using a standard protocol and imaged using an inverted microscope (Olympus, Japan) (Alberton et al., 2015).

### Chondrogenesis: GAG Assay and Histological Staining

Hyperoxia and physioxia expanded crude meniscus cells, avMPCs, and vMPCs were used to form pellet cultures as previously described (Pattappa et al., 2019b). In brief, pellets were formed by centrifuging 2 × 10^5^ cells at 250× *g* for 5 min in 300 µl of chondrogenic medium in polypropylene V-bottom 96-well plates. Chondrogenic media consisted of serum-free high-glucose DMEM containing 10 ng/ml TGF-β1 (R&D Systems), 100 nM dexamethasone, 50 μg/ml ascorbic acid-2-phosphate (all Sigma-Aldrich, Steinheim, Germany), 1 mM sodium pyruvate (Invitrogen, Karlsruhe, Germany), and 1% ITS (PAN Biotech GmbH, Aidenbach, Germany). Media changes were performed three times per week and then collected for analysis after 21 days.

Pellets were collected for wet weight and GAG assay using protocols previously described (Pattappa et al., 2019b). A set of pellets were also fixed in 4% PBS buffered paraformaldehyde, rinsed briefly in PBS, and then incubated in increasing sucrose concentrations (10%–30%). Pellets were photographed with an optical microscope (PL 2000, Optech, Germany) and then embedded in Tissue-Tek (Sakura, Zoeterwnde, Netherlands). Cryosections (10 µm thick) were created using a cryotome (HM500 OM; Microm, Berlin, Germany) and then stained with DMMB for visualization of glycosaminoglycans using previously published protocols (Pattappa et al., 2019b; Pattappa et al., 2020).

Sections were immunohistochemically labeled for human type I collagen (mouse monoclonal antibody, 1:50; C256; Sigma-Aldrich), human type II collagen (mouse monoclonal antibody, 1:200; CP18, Calbiochem, Darmstadt, Germany), and type X collagen (mouse monoclonal antibody, 1:50; X53, ThermoFisher Scientific), then labeled with a corresponding fluorescently labeled secondary antibody [tetramethylrhodamine (TRITC)-conjugated anti-mouse IgG (1:200; Jackson ImmunoResearch, Cambridge, UK)] using previously described protocols (Pattappa et al., 2019b). Sections were imaged on an Olympus XC10 camera on an Olympus BX61 fluorescence microscope (Olympus, Japan).

### Gene Expression Analysis

RNA was extracted from a pool of six pellets from crude avascular and vascular meniscus cells and avMPCs and vMPCs from each condition using previously described protocols (Pattappa et al., 2019b). Total RNA was quantified and 250 ng was reverse-transcribed using Transcriptor first strand kit (Roche, Mannheim, Germany). qPCR reactions for chondrogenic genes (see Supplementary Table S1) were performed using Brilliant SYBR Green QPCR mix (Stratagene, San Diego, CA, United States) and measured using a Biorad CFX96 system (Biorad Laboratories, Munich, Germany)**.** Results were analyzed using the ΔΔCt method with Proteasome subunit beta type-4 (PSMB4) used as a housekeeping gene and then presented as fold change under physioxia relative to hyperoxia for avMPCs, vMPCs, and crude meniscus cells (Pattappa et al., 2019b; Pattappa et al., 2020).

### Statistical Analysis

All statistical analysis was performed using GraphPad Prism v7 (GraphPad, La Jolla, CA, United States). Data were checked for normal distribution using a Shapiro-Wilk test. A comparison of pellet wet weight and GAG content between hyperoxia and physioxia was performed using two-way ANOVA with Tukey post-hoc test, with significance set at *p* < 0.05. Gene expression data were calculated as a fold change in physioxia relative to hyperoxia and then analyzed using a Mann–Whitney test with significance set at *p* < 0.05.

## Results

### Non-Fibronectin Adherent Vascular Meniscus Cells Contain a Progenitor Population With Trilineage Differentiation Potential

**FIGURE 2:**
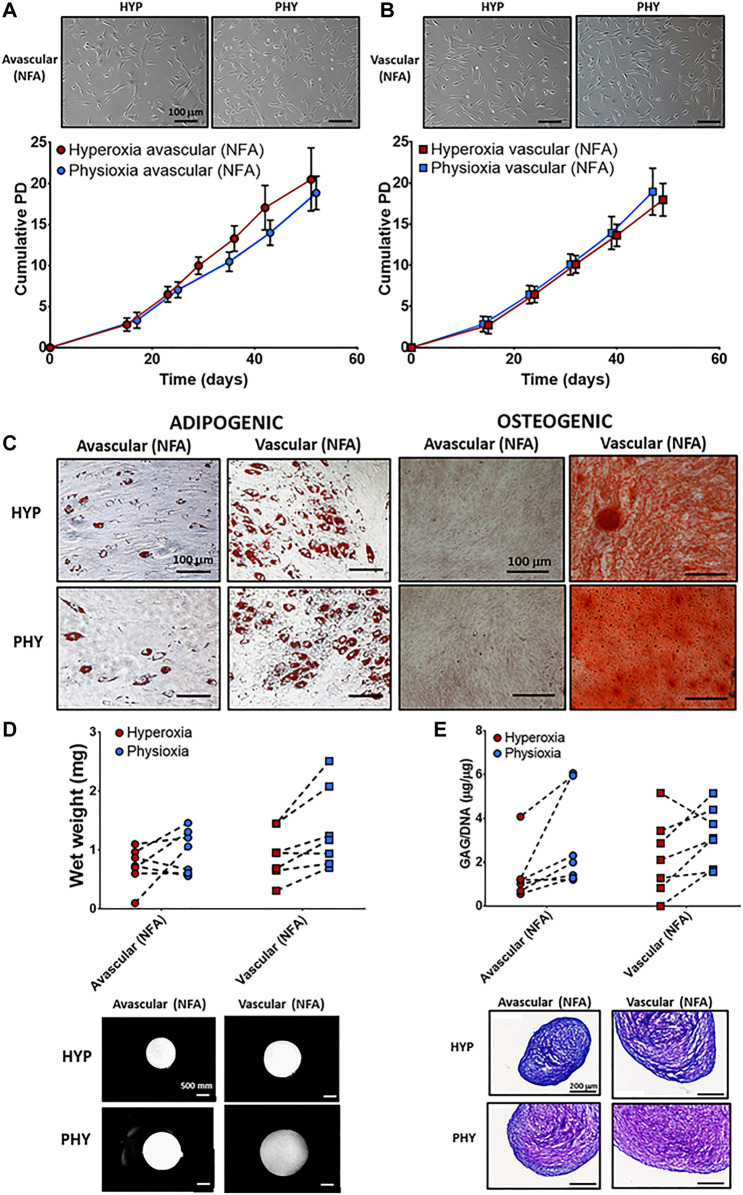
Representative photomicrographs of hyperoxia (HYP) and physioxia (PHY) expanded NFA **(A)** avascular and **(B)** vascular meniscus cells with corresponding population growth curves (*n* = 7; mean ± S.D.). **(C)** Representative photomicrographs of Oil-red-O staining for lipid droplet and alizarin red staining for calcium deposition for meniscus populations under hyperoxia (HYP) and physioxia (PHY). Pellet **(D)** wet weight **(E)** GAG content for avascular and vascular meniscus cells with representative **(D)** macroscopic and **(E)** DMMB-stained pellets.

No differences were observed in cellular morphology between NFA avascular (Figure 2A) and vascular (Figure 2B) meniscus cells under hyperoxia and physioxia. There was no difference in cumulative cell doublings between NFA avascular and vascular meniscus cells expanded under hyperoxia or physioxia (Figures 2A,B). Lipid droplet formation was observed for both meniscus populations (NFA) independent of oxygen tension with avascular meniscus cells (NFA) observed to have fewer lipid vacuoles compared to vascular meniscus cells (Figure 2C). In contrast, osteogenic differentiation of meniscus cells showed only positive alizarin red staining for vascular meniscus cells (NFA) under both oxygen conditions, whereas avascular meniscus cells showed no alizarin red staining independent of oxygen tension (Figure 2C).

There was no significant difference in pellet wet weight and GAG deposition between meniscus regions under different oxygen tension for chondrogenic differentiation (Figures 2D,E). Examination of individual donors demonstrated that 70% of donors had an increase in pellet wet weight and GAG deposition under physioxia compared to hyperoxia for both meniscus cell types (Figures 2D,E). Macroscopic images and DMMB staining of a donor that was physioxia responsive showed that avascular and vascular meniscus pellets appeared larger and had greater metachromasia staining compared to corresponding hyperoxia pellets (Figures 2D,E). There was an upregulation in SOX9, COL2A1, COL1A1, and ACAN under physioxia for both meniscus regions, while there was a downregulation in COL10A1 under physioxia for vascular meniscus cells (Supplementary Figure S1A). Increased type I collagen and type II collagen staining for avascular and vascular meniscus cells was observed under physioxia, while there was no staining for collagen X for both regions independent of oxygen tension (Supplementary Figure S1B).

### Fibronectin Adherence and Physioxia Isolates a Progenitor Population From the Avascular and Vascular Regions of Meniscus With High Clonogenic and Multilineage Differentiation Capacity

**FIGURE 3:**
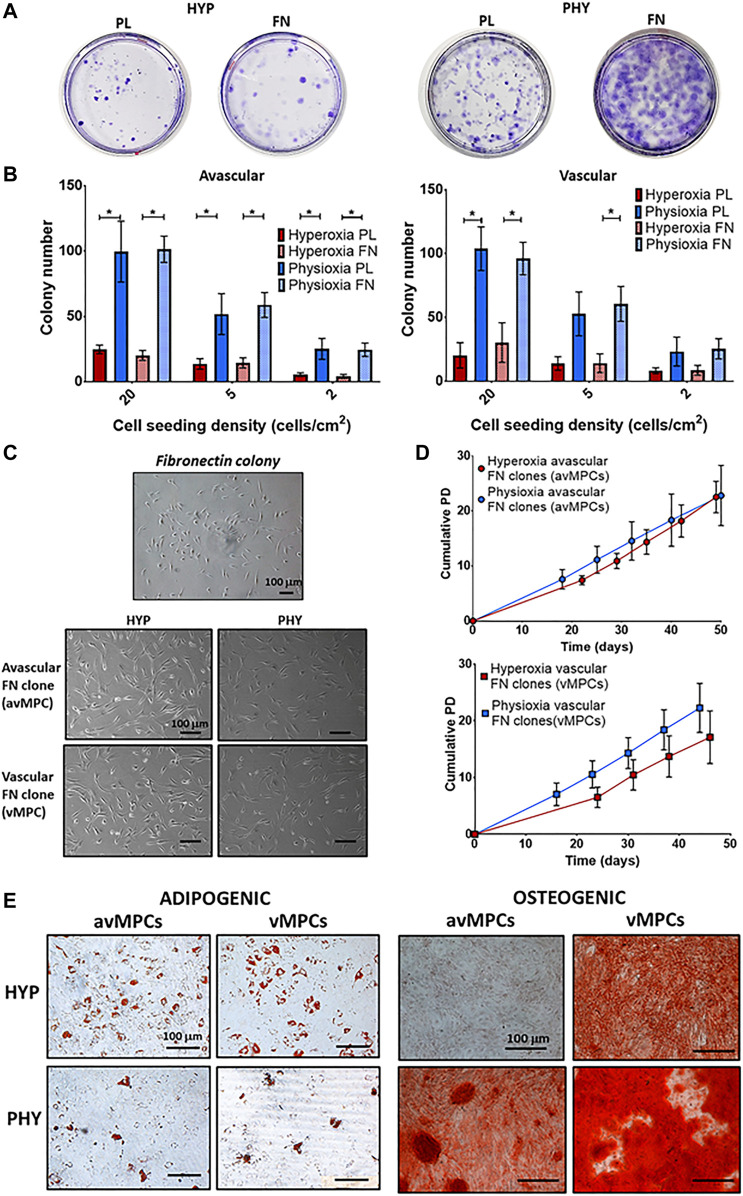
**(A)** Representative crystal violet-stained colonies cultured under hyperoxia (HYP) and physioxia (HYP) on uncoated (PL) and fibronectin (FN)-coated dishes. **(B)** Number of colonies counted from conditions described in **(A)** from avascular and vascular meniscus cells (*n* = 6; data represent mean ± S.D.; *p < 0.05). **(C)** Representative photomicrographs of a fibronectin colony and avMPCs and vMPCs cultured under hyperoxia and physioxia with **(D)** population growth curves for expanded avMPC and vMPC population under hyperoxia and physioxia [*n* = 4 (3 clones/donor); data represent mean ± S.D.]. **(E)** Representative Oil-red-O staining for lipid droplet formation and alizarin red staining for calcium deposition for avMPCs and vMPCs.

CFU-F evaluation showed that physioxic cultures had increased colony formation compared to hyperoxic cultures, with fibronectin adherent colonies observed to have larger diameter colonies compared to those formed on uncoated plates (Figure 3A). Physioxia significantly increased colony number for avascular and vascular meniscus cells on both plastic and fibronectin-coated dishes compared to corresponding dishes cultured under hyperoxia (*p < 0.05; Figure 3B). Furthermore, a similar pattern was observed for the CFU-F efficiency, whereby a higher efficiency was observed under physioxia with a tendency towards increased CFU-F efficiency upon combining physioxia and fibronectin (*p < 0.05; Supplementary Figure S2). There was no difference in avMPCs and vMPCs cell morphology between oxygen conditions (Figure 3C). Specifically, physioxic vMPCs appeared to have greater cumulative population doublings compared to equivalent hyperoxic vMPCs (Figure 3D). However, no significant difference was observed between cumulative population doublings or doubling time at each passage under physioxia compared to hyperoxia for avMPCs or vMPCs (Figure 3D). Adipogenesis was exhibited by both hyperoxic and physioxic avMPCs and vMPCs with greater lipid vacuoles formed for hyperoxic avMPCs and vMPCs (Figure 3E). In contrast, alizarin red staining for osteogenesis indicated a mineralized matrix by hyperoxic vMPCs, while both physioxic avMPCs and vMPCs also stained for alizarin red (Figure 3E).

### Physioxia Isolated avMPCs and vMPCs Show Enhanced Matrix Capacity With Minimal Collagen X Expression

**FIGURE 4:**
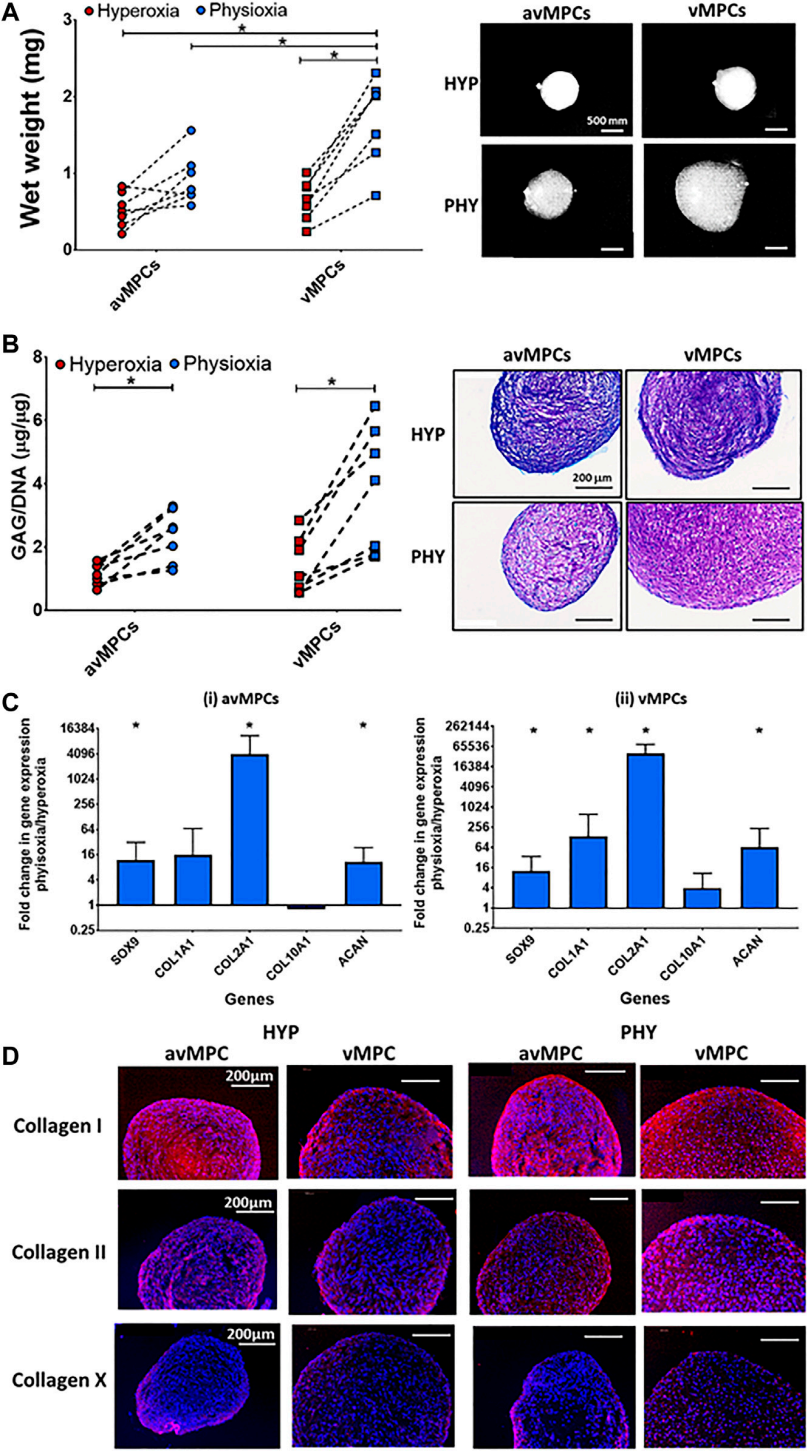
Chondrogenic differentiation of avMPCs and vMPCs under hyperoxia and physioxia. Dot plot for pellet **(A)** wet weight and **(B)** GAG content for isolated clones (each dot represents mean of individual three clones) cultured under hyperoxia and physioxia with representative **(A)** macroscopic and **(B)** DMMB-stained pellets. **(C)** Gene expression data for meniscus matrix genes for (i) avascular and (ii) vascular MPCs. Data represent fold change for (i) avascular and (ii) vascular MPCs cultured under physioxia relative to corresponding clones under hyperoxic conditions (data represent mean ± S.D.; *n* = 5 donors; *p < 0.05). **(D)** Representative images of avMPC and vMPCs pellets stained with collagen I, collagen II, and collagen X cultured under hyperoxia and physioxia. Positive and negative controls are in Supplementary Figure S1.

Physioxia vMPCs showed a significant increase in both wet weight (*p < 0.05; Figure 4A) and GAG deposition (*p < 0.05; Figure 4B) compared to hyperoxic vMPCs. Physioxic avMPCs had both an increase in wet weight and a significant increase in GAG content compared to hyperoxic avMPCs (*p < 0.05; Figure 4B). Macroscopic images and DMMB staining showed larger pellets and greater DMMB staining formed for physioxia meniscus progenitors (Figures 4A,B).

A significant upregulation in SOX9, and matrix genes COL2A1, COL1A2, and ACAN was observed for physioxic avMPCs or vMPCs compared to corresponding hyperoxic progenitors (*p < 0.05; Figure 4C). An increase in type I and II collagen staining was also observed for physioxia avMPCs and vMPCs compared to corresponding hyperoxic progenitors (Figure 4D). Furthermore, there was no collagen X staining for progenitors cultured under either oxygen condition (Figure 4D).

## Discussion

The present study has demonstrated that a progenitor population can be isolated *via* combined differential fibronectin adhesion and physioxia from the avascular and vascular regions of the meniscus with enhanced clonogenicity and differentiation potential of these populations (Figure 3). Similar to previous studies using ACPs, physioxic culture of avMPCs or vMPCs demonstrated an increase in matrix content by these cells with minimal presence of collagen X (Figure 4) (Anderson et al., 2016). In comparison, examination of non-fibronectin adherent meniscus population only indicated that the vascular region of the meniscus contained a population with trilineage differentiation potential with no difference in cell expansion between oxygen tensions (Figure 2).

Previous studies have described the presence of multipotent meniscus cell populations from either animal sources (e.g., bovine), human meniscus tissue, or debris (Mauck et al., 2007; Muhammad et al., 2014; Gamer et al., 2017; Liang et al., 2017; Seol et al., 2017; Sun et al., 2020; Chahla et al., 2021; Wei et al., 2021). Specifically, the horns and vascular regions of the tissue appear to contain progenitor populations (Mauck et al., 2007). However, few studies have investigated their differentiation potential under physioxia. Liang et al. (2017) showed that physioxia enhanced and maintained the adipogenic and chondrogenic potential of meniscus cells for up to 10 population doublings, with no osteogenesis observed under both oxygen tensions. However, there was no separation between regions in the stated study. The results of the non-fibronectin adherent meniscus cells suggest that the vascular region of the meniscus contained a population with trilineage differentiation potential under both oxygen conditions similar to the previously stated investigations (Figure 2). The results of the chondrogenic data showed a donor-dependent increase in matrix deposition for meniscus cells under physioxia, similar to recent studies for chondrocytes or chondrogenic MSCs (Anderson et al., 2016; Pattappa et al., 2019a; Pattappa et al., 2019b). An upregulation in COL1A1 and COL2A1 expression and greater collagen I and II staining was observed for avascular and vascular meniscus cells (NFA) under physioxia compared to hyperoxia expanded cells, similar to results from previous studies (Supplementary Figure S1) (Adesida et al., 2006; Adesida et al., 2007; Berneel et al., 2016).

The second part of this study combined physioxia with differential fibronectin adherence to understand whether progenitor populations can be directly isolated from both avascular and vascular regions of the meniscus using this method (Figures 3, 4). Isolation using differential fibronectin adherence for articular cartilage yielded a population with increased telomerase activity that enabled it to undergo high population doublings with maintenance of their differentiation capacity in both healthy and osteoarthritic cartilage (Dowthwaite et al., 2004; Williams et al., 2010). A recent investigation demonstrated that from OA meniscus tissue, fibronectin adherence enabled the isolation of a progenitor population that maintained both its clonogenicity and multipotency at high passages with expression of stem cell markers (e.g. CD90 and CD105) compared to non-fibronectin adherent meniscus cells (Korpershoek et al., 2021). In contrast to the stated study and the results from the non-fibronectin adherent meniscus cells, the present study demonstrated that the combined use of differential fibronectin adherence and physioxia yielded a progenitor population from both the avascular and vascular meniscus regions with trilineage differentiation capacity (Figures 3, 4). This result supports the results of recent investigations that have demonstrated the presence of progenitor populations in avascular regions of the meniscus (Muhammad et al., 2014; Chahla et al., 2021). Additionally, the presence of fibronectin has a tendency to increase CFU-F efficiency of avascular and vascular meniscus cells under physioxia, indicating that fibronectin could efficiently isolate meniscus progenitor populations (Supplementary Figure S2).

It should be noted that the osteogenic lineage demonstrated variable differentiation among the avMPCs and vMPCs examined, particularly upon physioxic culture. Previous publications have contrasting data regarding whether physioxia either inhibits (D'Ippolito et al., 2006) or enhances (Wagegg et al., 2012; Yu et al., 2019) osteogenic differentiation. Our results show that the majority of clonal progenitors studied differentiated to the osteogenic lineage under physioxia. However, there were also progenitors with no osteogenic induction that may be related to physioxia inhibiting osteogenesis. Furthermore, this could also be related to inherent multipotency of the specific clonal progenitor. Loncaric et al. (2019) demonstrated that even within MSC cultures, only 40% of clonal populations derived from a single cell exhibit multipotency and sustain their clonogenicity at high passages (Loncaric et al., 2019). Thus, further clonal progenitor isolation would need to be performed for fibronectin adherent meniscus cells (avMPCs and vMPCs) with single cells taken from these clones to understand whether these are true multipotent progenitors. Additionally, this would also show whether the inhibited osteogenesis for avMPCs and vMPCs under physioxia was due to the oxygen tension or inherent differentiation capacity.

To demonstrate that the avMPCs and vMPCs are progenitor populations, examination of their cell surface marker expression needs to be performed. Single-cell RNAseq analysis has identified progenitor populations within healthy and osteoarthritic meniscus (Sun et al., 2020). They showed that a CD146-positive population had progenitor characteristics within healthy meniscus, while in osteoarthritic meniscus, this changed to a CD318-positive population *via* attenuation of TGF-β-related pathways (Sun et al., 2020). CD146-positive cells are termed pericytes and found to be present near vasculature (e.g., bone marrow) and have stem cell characteristics (Crisan et al., 2008). Korpershoek et al. (2021) showed a significant increase in CD318 expression in their fibronectin adherent meniscus progenitors compared to non-fibronectin-adherent meniscus cells, indicating that these are dervied from osteoarthritic meniscus. Future investigations would study cell surface markers from the populations described in the present study.

The use of physioxia significantly enhanced the clonogenicity of the meniscus population with fibronectin producing colonies with larger diameter (Figures 3A,B). This is potentially related to the stem cell property of cellular migration that assists in self-renewal and cellular proliferation and has been known to be enhanced upon culture on fibronectin (Singh and Schwarzbauer, 2012). The subsequent growth under both oxygen conditions demonstrates that the avMPCs and vMPCs under both oxygen conditions have the potential to be expanded to high population doublings with no discernible changes in doubling time or differentiation potential (Figure 3D). Further analysis is required to see whether their differentiation potential remains the same with passage for this clonal population compared to non-fibronectin adherent meniscus cells (Korpershoek et al., 2021). However, in spite of combined fibronectin and physioxia isolating progenitors from both meniscus regions, there remains an open question regarding the additional benefit of fibronectin towards creating a cell-based therapy for meniscus regeneration. Previous studies have shown that physioxia helps maintain the stemness and expression of stem cell genes (D'Ippolito et al., 2006), whilst fibronectin adherent progenitors have demonstrated similar properties with the additional benefit of increased telomerase activity compared to non-fibronectin adherent populations (Dowthwaite et al., 2004; Williams et al., 2010). Due to the similarities in their effect on progenitor populations, future investigations would seek to understand the specific effects of fibronectin on isolation of meniscus progenitors both on its own and in combination with physioxia. Furthermore, this would help justify the use of fibronectin for meniscus progenitor isolation, as this step adds additional costs towards the creation of cell-based therapies for meniscus treatment.

Physioxic culture of avMPCs and vMPCs had a significant increase in cartilage matrix genes, GAG deposition, and collagen I/II staining for both meniscus regions compared to hyperoxic cultures (Figure 4). Furthermore, similar to findings for ACPs, no collagen X staining was found in these progenitor populations, independent of oxygen tension (Anderson et al., 2016)**.** A limitation of bone marrow MSCs for chondrogenesis is that it expresses markers for hypertrophy *in vitro* and forms bone upon *in vivo* implantation, inspite of physioxic expansion (Pelttari et al., 2006; Pattappa et al., 2019b). Thus, the present results demonstrate the importance of physioxia for the induction of a meniscus phenotype, specifically for matrix deposition and preventing collagen X expression that could lead to hypertrophy *in vivo*. Future studies would evaluate the feasibility of these meniscus progenitors for clinical translation by isolating progenitors from animal meniscus and then test their healing potential in treating either meniscal tears or pars intermedia defects (Angele et al., 2008; Koch et al., 2019). Additionally, the presence of these progenitor populations provides an additional rationale for the development of biomaterial-based technologies that allow cellular migration *via* suitable chemoattractants, which enables natural healing of meniscus tears without the necessity for extensive cell culture (Qu et al., 2017).

## Conclusion

Physioxia enhances the clonogenicity of avascular and vascular meniscus cells, with larger diameter colonies formed on fibronectin. Combined physioxia and fibronectin adherence isolated a progenitor population with trilineage differentiation potential from the avascular and vascular meniscus regions. Specifically for meniscus tissue engineering, these progenitor cells were observed to have enhanced matrix content under physioxia with suppression of collagen X that could make them less susceptible to hypertrophy upon *in vivo* implantation. Therefore, combined differential fibronectin adherence and physioxia is a potential method for isolating progenitor populations from the meniscus that can be used for the development of cell-based therapies for the treatment of meniscus tears/defects.

## Data Availability

The original contributions presented in the study are included in the article/Supplementary Material. Further inquiries can be directed to the corresponding author.
