# Supplementary material for: Fibronectin Adherent Cell Populations Derived From Avascular and Vascular Regions of the Meniscus Have Enhanced Clonogenicity and Differentiation Potential Under Physioxia
Source: Front Bioeng Biotechnol. 2022 Jan 28;9:789621. doi: 10.3389/fbioe.2021.789621 (PMC8831898; doi:10.3389/fbioe.2021.789621)
Supplement: Supplementary file 1 [file Table1.DOCX]

| **Gene** | **Sequence (Forward)** | **Sequence (Reverse)** | **NCBI Accession Number** |
| --- | --- | --- | --- |
| *PSMB4* | GCTTAGCACTGGCTGCTTCT | GGACATGCTTGGTGTAGCCT | NM_002796.3 |
| *SOX9* | ACACACAGCTCACTCGACCTTG | AGGGAATTCTGGTTGGTCCTCT | NM_000346.4 |
| *COL1A1* | ACGTCCTGGTGAAGTTGGTC | ACCAGGGAAGCCTCTCTCTC | NM_000088.4 |
| *COL2A1* | GGGCAATAGCAGGTTCACGTA | TGTTTCGTGCAGCCATCCT | NM_033150.3 |
| *COL10A1* | CCCTCTTGTTAGTGCCAACC | AGATTCCAGTCCTTGGGTCA | NM_000493.4 |
| *ACAN* | GGCACTTCAGTTGCAGAAGG | CTATACCCCAGTGGGCACAT | NM_001135.4 |

**Supplementary table 1: Genes and primer sequences for evaluation of meniscogenic pellets**
