# Supplementary figures and images for: Fibronectin Adherent Cell Populations Derived From Avascular and Vascular Regions of the Meniscus Have Enhanced Clonogenicity and Differentiation Potential Under Physioxia
Source: Front Bioeng Biotechnol. 2022 Jan 28;9:789621. doi: 10.3389/fbioe.2021.789621 (PMC8831898; doi:10.3389/fbioe.2021.789621)

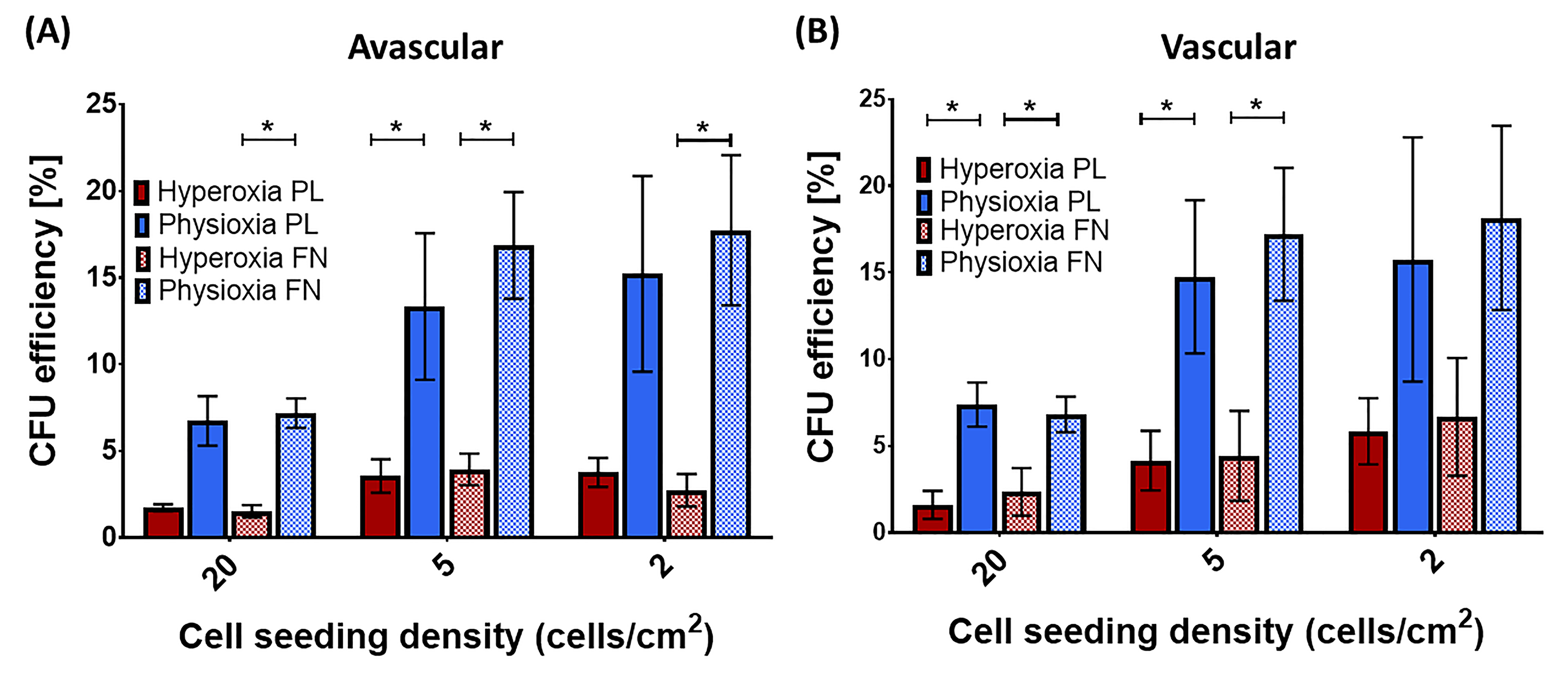

Supplement: Supplementary file 2 [file Image2.TIF]

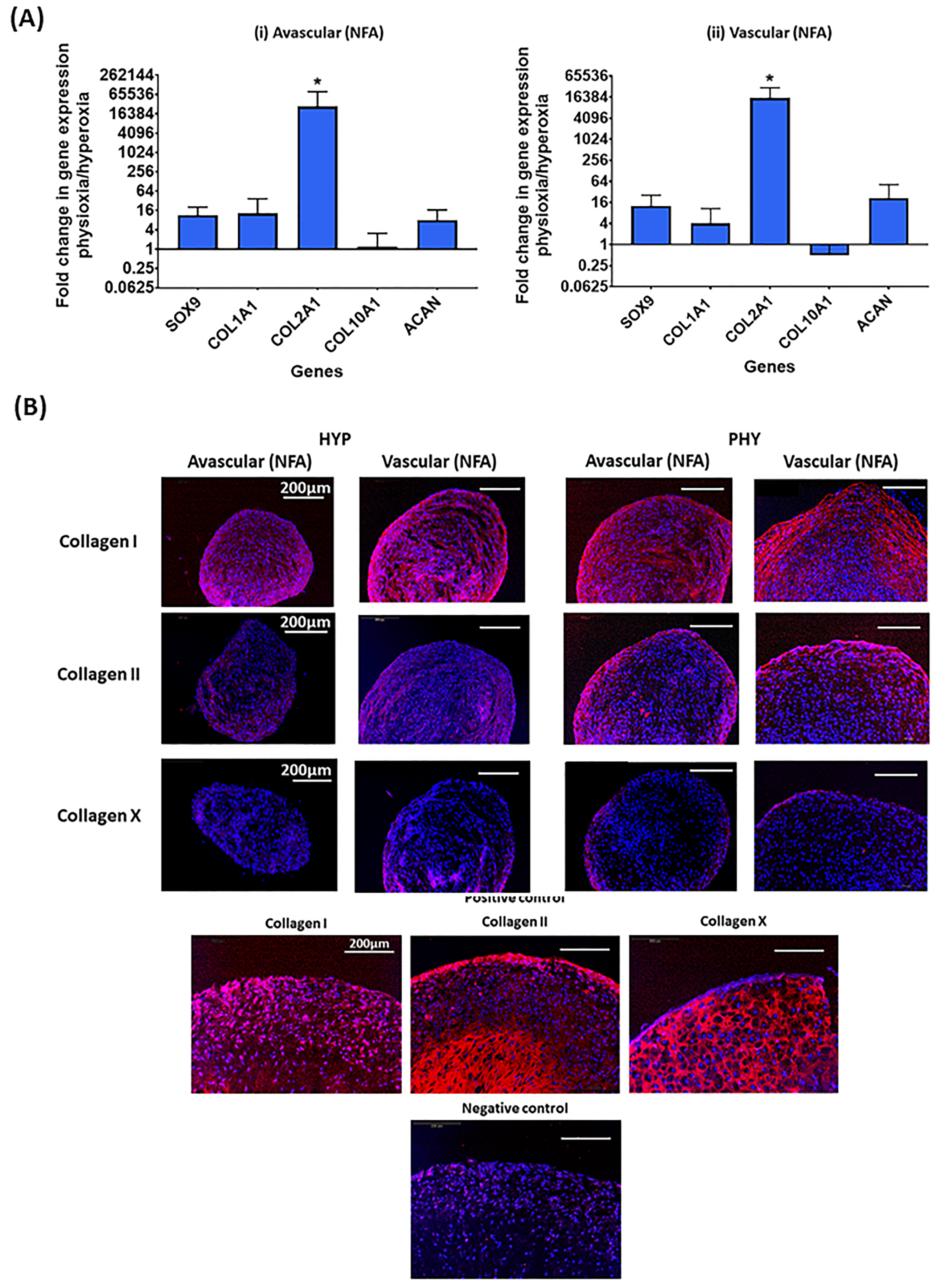

Supplement: Supplementary file 3 [file Image1.TIF]
