# Supplementary material for: Fibronectin Adherent Cell Populations Derived From Avascular and Vascular Regions of the Meniscus Have Enhanced Clonogenicity and Differentiation Potential Under Physioxia
Source: Front Bioeng Biotechnol. 2022 Jan 28;9:789621. doi: 10.3389/fbioe.2021.789621 (PMC8831898; doi:10.3389/fbioe.2021.789621)
Supplement: Supplementary file 4 [file DataSheet1.docx]

**Supplementary figure 1**. Gene expression data for meniscus matrix genes for (i) avascular and (ii) vascular meniscal cells. Data represent fold change of crude (i) avascular and (ii) vascular meniscal cells cultured under physioxia relative to corresponding hyperoxic meniscal cells (data represent mean + S.D.; n = 5 donors; *p < 0.05). (d) Representative images of avascular and vascular meniscal pellets stained collagen I, collagen II and collagen X cultured under hyperoxia and physioxia. Positive control for each antibody stain are bone marrow MSC pellets cultured in chondrogenic media under hyperoxia.

**Supplementary figure 2.** Colony-forming unit assay efficiency (%) for colonies formed under hyperoxia (HYP) and physioxia (PHY) on uncoated (PL) and fibronectin (FN) coated dishes for avascular and vascular meniscus cells (n = 6; data represents mean + S.D.; *p < 0.05).

**Supplementary table 1.** Genes and primer sequences for evaluation of meniscogenic pellets
